# Antioxidant Defense, Lignin Biosynthesis and Hormone Signaling Coordinately Mediate the Response of *Solanum tuberosum* L. to Alkaline Salt Stress

**DOI:** 10.3390/plants15213377

**Published:** 2026-11-01

**Authors:** Mingyang Hu, Yichen Kang

**Affiliations:** 1 College of Food Science and Engineering, Gansu Agricultural University, Lanzhou 730070, China; huganane@163.com; 2 College of Horticulture, Gansu Agricultural University, Lanzhou 730070, China

**Keywords:** potato, alkaline salt stress, antioxidant system, lignin, plant hormones, NaHCO_3_

## Abstract

Alkaline salt stress is an important abiotic stress factor that restricts the growth and development of potato (*Solanum tuberosum* L.). It can induce oxidative damage, disrupt cellular structures, and disturb endogenous hormone homeostasis, thereby impairing normal plant growth and development. However, the physiological mechanisms underlying potato responses to alkaline salt stress, as well as the relationships among antioxidant defense, lignin accumulation, and changes in endogenous hormone levels, remain to be fully elucidated. Therefore, a systematic investigation of potato responses to alkaline salt stress at both the physiological and gene expression levels is needed to provide further insights into the underlying stress-response mechanisms. In this study, potato cultivar ‘Atlantic’ was used as the experimental material, and a pot experiment was conducted with four NaHCO_3_ concentrations: 0 mmol/L (CK), 20 mmol/L (T1), 40 mmol/L (T2), and 60 mmol/L (T3). Changes in the antioxidant system, root lignin accumulation, and endogenous hormone contents under different levels of alkaline salt stress were quantitatively analyzed. In addition, the expression patterns of genes involved in lignin biosynthesis (phenylpropanoid pathway) and ABA, GA, and BR signaling pathways were examined to establish an integrated model of the responses involving antioxidant defense, hormone regulation, and lignin biosynthesis. The results showed that the degree of oxidative stress progressively increased with increasing NaHCO_3_ concentration. SOD (superoxide dismutase) activity and the contents of MDA (malondialdehyde) and GSH (glutathione) continuously increased, whereas POD(peroxidase) and CAT (catalase) activities exhibited a pattern of first increasing and then decreasing, reaching their maximum levels under 40 mmol/L NaHCO_3_ treatment. Root lignin content increased progressively with increasing stress intensity. In addition, alkaline salt stress significantly affected endogenous hormone levels, with ABA and BR contents increasing and GA content decreasing as the NaHCO_3_ concentration increased. Further analysis revealed that alkaline salt stress altered the expression of genes involved in the phenylpropanoid pathway and ABA (abscisic acid), GA (gibberellin), and BR (brassinosteroid) signaling pathways, indicating coordinated responses involving antioxidant defense, lignin biosynthesis, and hormonal regulation. In conclusion, potato responds to alkaline salt stress by enhancing antioxidant defense, promoting lignin accumulation, and modulating endogenous hormone balance, which may contribute to its adaptation to alkaline salt stress. This study provides insights into the regulatory mechanisms underlying potato responses to alkaline salt stress and provides a theoretical basis for stress-resilient potato cultivation and the breeding of salt- and alkali-tolerant cultivars in saline-alkali soils.

## 1. Introduction

Potato (*Solanum tuberosum* L.) is an annual herbaceous plant belonging to the Solanaceae family. It is the fourth-largest staple food crop in the world and the most important non-cereal food crop globally [1]. Owing to its tolerance to saline-alkali conditions and drought, strong adaptability, and high nutritional value, including abundant carbohydrates, amino acids, vitamins, and minerals, potato is cultivated in more than 100 countries worldwide and is consumed by more than one billion people, with an annual production exceeding 300 million tons [2,3]. China is the largest producer and consumer of potato worldwide [4]. In recent years, China has actively promoted the potato-as-a-staple-food strategy, which aims to facilitate structural reform of the agricultural supply side and alleviate pressures on national food security [5]. With the development of modern agriculture, factors such as inappropriate cultivation practices, excessive fertilizer application, and over-irrigation have contributed to the increasing severity of soil salinization and alkalization in China, posing a serious threat to potato production [6,7]. In particular, large areas of saline-alkali land are distributed in northwestern China [8]. Therefore, investigating the effects of alkaline salt stress on the antioxidant and oxidative damagesystems, lignin accumulation, and phytohormone levels in potato, as well as identifying approaches to alleviate the adverse effects of salinity and alkalinity on potato cultivation, is of great significance for improving potato yield and quality.

China has a vast land area; however, the proportion of cultivated land affected by saline-alkali stress has been increasing in recent years [9,10,11]. In China, saline-alkali stress is considered the second most important abiotic stress limiting agricultural production. Soil salinization can disrupt cellular ion homeostasis, impair cell membrane structure, and interfere with plant water uptake, ultimately resulting in reduced crop productivity [12,13]. Unsustainable cultivation practices, excessive fertilizer use, and, in particular, over-irrigation are major factors contributing to the salinization and alkalization of cultivated soils [14,15]. Compared with neutral salt stress, alkaline salt stress causes more severe damage to soils, particularly in northwestern China, where alkaline salts represented by NaHCO_3_ cause substantial soil degradation [16,17]. Because saline–alkali soils in China are characterized by their extensive distribution and large spatial fragmentation, they pose a considerable threat to agricultural production [18,19]. Therefore, elucidating the mechanisms underlying plant responses to saline-alkali stress is of considerable practical importance.

During plant responses to alkaline salt stress, oxidative damage, structural defense, and phytohormone-mediated signaling collectively contribute to adaptation to adverse environmental conditions [20,21]. Alkaline salt stress induces excessive accumulation of reactive oxygen species (ROS), resulting in oxidative stress and membrane lipid peroxidation [22]. Plants therefore activate antioxidant defense systems to maintain cellular redox homeostasis. Among these systems, superoxide dismutase (SOD) serves as the first line of defense against ROS by catalyzing the dismutation of superoxide anion radicals into hydrogen peroxide [23]. Peroxidase (POD) and catalase (CAT) subsequently participate in the decomposition of hydrogen peroxide, thereby reducing the extent of oxidative damage [24]. Malondialdehyde (MDA), a major product of membrane lipid peroxidation, is widely used as an indicator of cell membrane damage [25]. Reduced glutathione (GSH), an important non-enzymatic antioxidant, can directly scavenge free radicals and maintain the intracellular reducing environment [26]. In addition, lignin, an essential component of the plant cell wall, plays an important role in structural defense under saline-alkali stress [27]. Under stress conditions, plants can activate the phenylpropanoid pathway to promote lignin biosynthesis and accumulation, thereby increasing cell wall mechanical strength, restricting the transport of harmful ions, and reducing water loss, which collectively enhance plant adaptation to saline-alkali environments [28]. Phytohormones, as important signaling molecules involved in the regulation of stress responses, also participate in the balance between growth and defense under alkaline salt stress [29]. Among them, abscisic acid (ABA) is a central hormone involved in plant responses to abiotic stress and can induce the expression of stress-responsive genes and enhance antioxidant capacity. Gibberellin (GA) primarily promotes plant growth and development, and changes in its levels under stress conditions contribute to the regulation of resource allocation between growth and defense [30]. Brassinosteroid (BR) can enhance plant stress tolerance by increasing antioxidant system activity and maintaining cell membrane stability [31]. Therefore, the coordinated regulation of the antioxidant system, lignin metabolism, and phytohormone signaling network constitutes an important physiological basis for plant adaptation to alkaline salt stress.

Previous studies have mainly focused on the effects of exogenous 24-epibrassinolide (EBR) on antioxidant defense, photosynthesis, and yield in potato under NaHCO_3_ stress, highlighting the role of hormone regulation in enhancing alkaline salt stress tolerance [30]. Unlike these studies, which primarily investigated the effects of exogenous hormone application, the present study comprehensively analyzed antioxidant-related parameters, root lignin accumulation, and the levels of endogenous abscisic acid (ABA), gibberellin (GA), and brassinosteroids (BR) in potato plants exposed to different concentrations of NaHCO_3_. In addition, the expression patterns of genes associated with phenylpropanoid metabolism and hormone signaling pathways were examined. By integrating physiological measurements with gene expression analysis, this study aimed to explore the relationships among the antioxidant defense system, root lignin accumulation, and changes in endogenous hormone levels under NaHCO_3_ stress, thereby providing a basis for understanding the associations between different physiological defense processes and their related molecular changes. Both conventional salt stress and alkaline salt stress can disrupt ion homeostasis and induce reactive oxygen species (ROS) accumulation in plants, thereby affecting antioxidant enzyme activities and lipid peroxidation. Previous studies have shown that changes in antioxidant enzyme activities under NaCl stress are associated with salt tolerance in potato cultivars [32]. Compared with conventional salt stress, alkaline salt stress involves not only osmotic stress and ion toxicity but also high-pH conditions and bicarbonate (HCO_3_^−^) stress [33]. In addition, the molecular responses of potato to alkaline salt stress have also been investigated in previous studies [34].

Lignin biosynthesis primarily occurs through the phenylpropanoid pathway. PAL, C4H, and 4CL catalyze key reactions in the initial stages of this pathway, contributing to the formation of cinnamic acid and its activated derivatives. HCT and C3′H are involved in the conversion of hydroxycinnamoyl esters and their hydroxylated derivatives. CCR catalyzes the reduction of hydroxycinnamoyl-CoA esters to the corresponding aldehydes, representing an important step in lignin monomer biosynthesis. CAD further catalyzes the reduction of cinnamaldehydes to the corresponding lignin monomer alcohols, whereas POD participates in the oxidative polymerization of lignin monomers, promoting lignin deposition in the cell wall. Therefore, changes in the expression of these genes may reflect responses in different stages of lignin biosynthesis and deposition [35]. Plant hormones are also involved in the regulation of stress responses. The abscisic acid (ABA) receptors PYR/PYL participate in ABA signal transduction by regulating SnRK2 kinases and ABF transcription factors [36]. DELLA proteins are important regulatory components of the gibberellin (GA) signaling pathway [37], whereas BRI1, a brassinosteroid (BR) receptor, mediates downstream signal transduction through components such as BSK [38]. Analyzing the expression patterns of genes associated with lignin biosynthesis and hormone signaling may therefore provide further insights into the physiological responses and molecular regulatory characteristics of potato under NaHCO_3_ stress.

In this study, potato cultivar ‘Atlantic’ was used as the experimental material, and different concentrations of NaHCO_3_ were applied to establish alkaline salt stress treatments. The effects of alkaline salt stress on the potato antioxidant system (SOD, POD, CAT, MDA, and GSH), root lignin accumulation, and endogenous phytohormone (ABA, GA, and BR) contents were systematically analyzed. Furthermore, expression analyses of key genes involved in lignin biosynthesis and phytohormone signaling pathways were conducted to further elucidate the physiological and molecular regulatory mechanisms underlying potato responses to alkaline salt stress. This study aimed to reveal the coordinated relationships among oxidative defense, structural defense, and phytohormone regulation during the response of potato to alkaline stress, thereby providing a theoretical basis for stress-resistant cultivation of potato in saline-alkali soils and the breeding of salt- and alkali-tolerant potato cultivars.

## 2. Results

### 2.1. Effects of Alkaline Salt Stress on the Antioxidant and Oxidative DamageSystems of Potato

**Figure 1:**
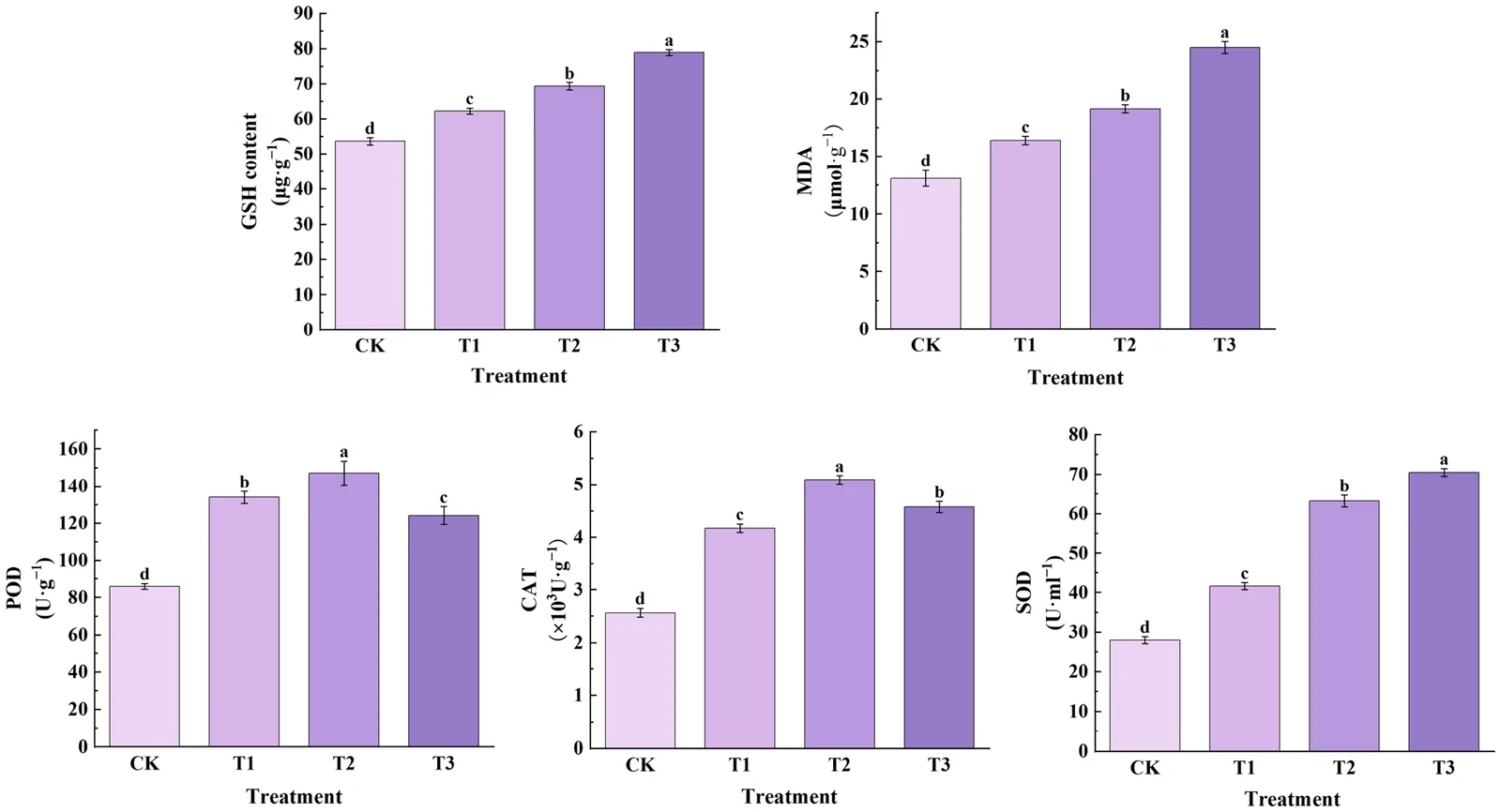
Effects of Alkaline Salt Stress on the Antioxidant System and Oxidative Damage in Potato Leaves. Note: CK, 0 mmol/L NaHCO_3_; T1, 20 mmol/L NaHCO_3_; T2, 40 mmol/L NaHCO_3_; and T3, 60 mmol/L NaHCO_3_. Data are presented as the mean ± standard error (SE) (*n* = 3). Different lowercase letters above the bars indicate significant differences among treatments (*p* < 0.05).

As shown in Figure 1, different levels of alkaline salt stress significantly affected the antioxidant and oxidative damagesystems of potato, and the measured parameters mainly showed an increasing trend with increasing stress severity. Specifically, with increasing sodium bicarbonate treatment concentration, SOD activity and GSH and MDA contents increased significantly, whereas POD and CAT activities first increased and then decreased. Among these parameters, the increases in SOD activity and MDA content were more pronounced, with SOD activity increasing more markedly at the early stage, while MDA content increased more markedly at the later stage. Compared with the control group (stress concentration of 0), SOD activity in the different treatment groups increased by 48.29%, 125.34%, and 151.35%, respectively. The higher the sodium bicarbonate concentration, the stronger the SOD activity. Compared with the control group, the T2 treatment showed the highest POD activity, which increased significantly by 71.20% compared with the control and was significantly higher than those in the T1 and T3 treatments. For CAT activity, the T2 treatment was also higher than the control group and was significantly higher than that of all other treatments. Compared with the control group (stress concentration of 0), MDA contents in the different treatment groups increased by 25.19%, 46.23%, and 87.07%, respectively, showing a gradual increase with increasing stress severity. For GSH content, the increase relative to the control group (stress concentration of 0) ranged from 15.93% to 47.12%. Compared with the increase in MDA content, the increase in GSH content was more uniform. The changes in both indicators indicated that the degree of stress experienced by potato plants increased with increasing stress severity.

### 2.2. Effects of Alkaline Salt Stress on Lignin Content in Potato

**Figure 2:**
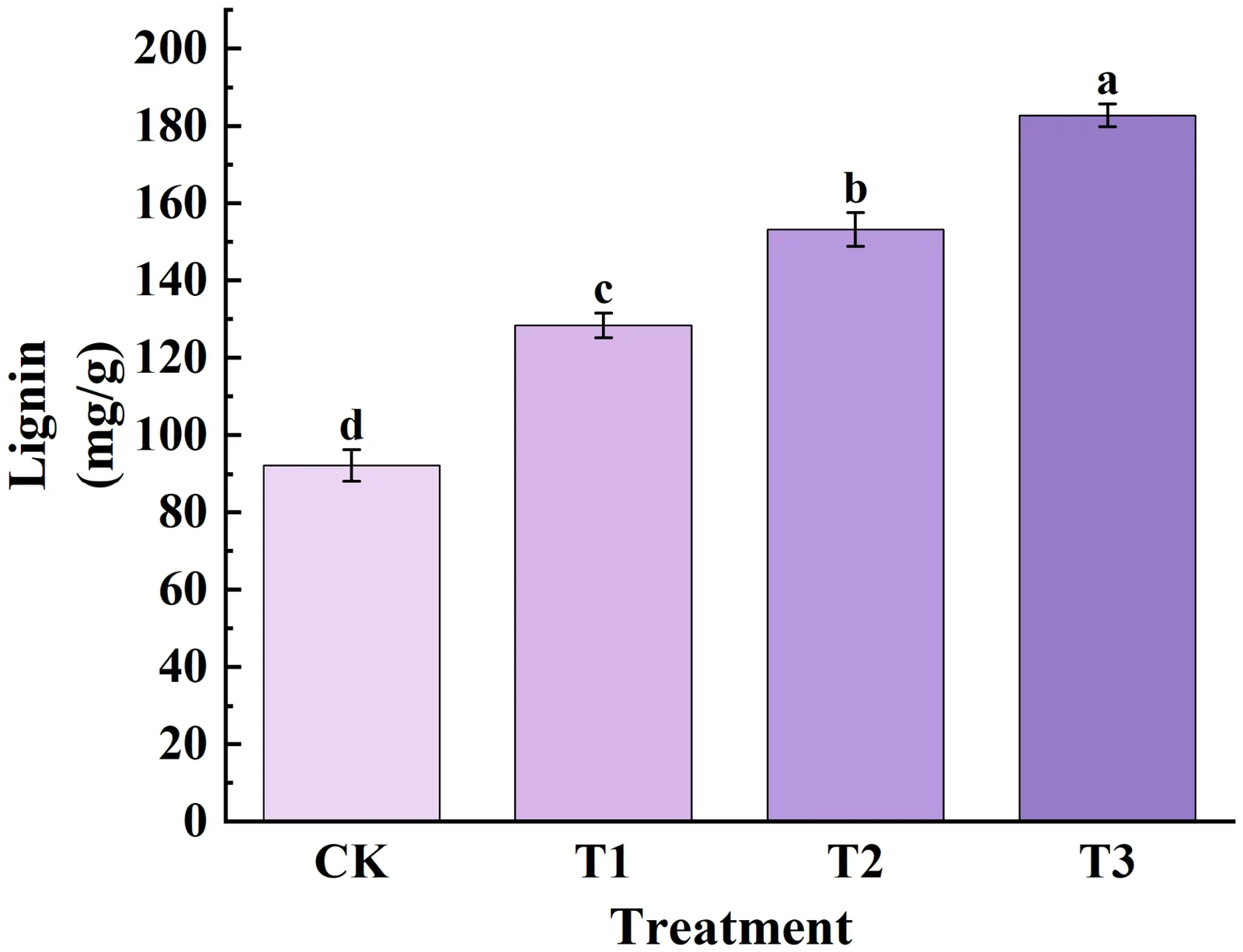
Effects of Alkaline Salt Stress on Root Lignin Content in Potato. Note: CK, 0 mmol/L NaHCO_3_; T1, 20 mmol/L NaHCO_3_; T2, 40 mmol/L NaHCO_3_; and T3, 60 mmol/L NaHCO_3_. Data are presented as the mean ± standard error (SE) (n = 3). Different lowercase letters above the bars indicate significant differences among treatments (*p* < 0.05).

As shown in Figure 2, lignin content gradually increased with increasing stress severity. Compared with CK, lignin content increased by 39.13%, 66.09%, and 98.19% in the different treatments, respectively. These results indicated that lignin content increased steadily with increasing sodium bicarbonate treatment concentration, thereby enhancing the stress resistance of potato plants.

### 2.3. Effects of Alkaline Salt Stress on Hormone Contents in Potato

**Figure 3:**
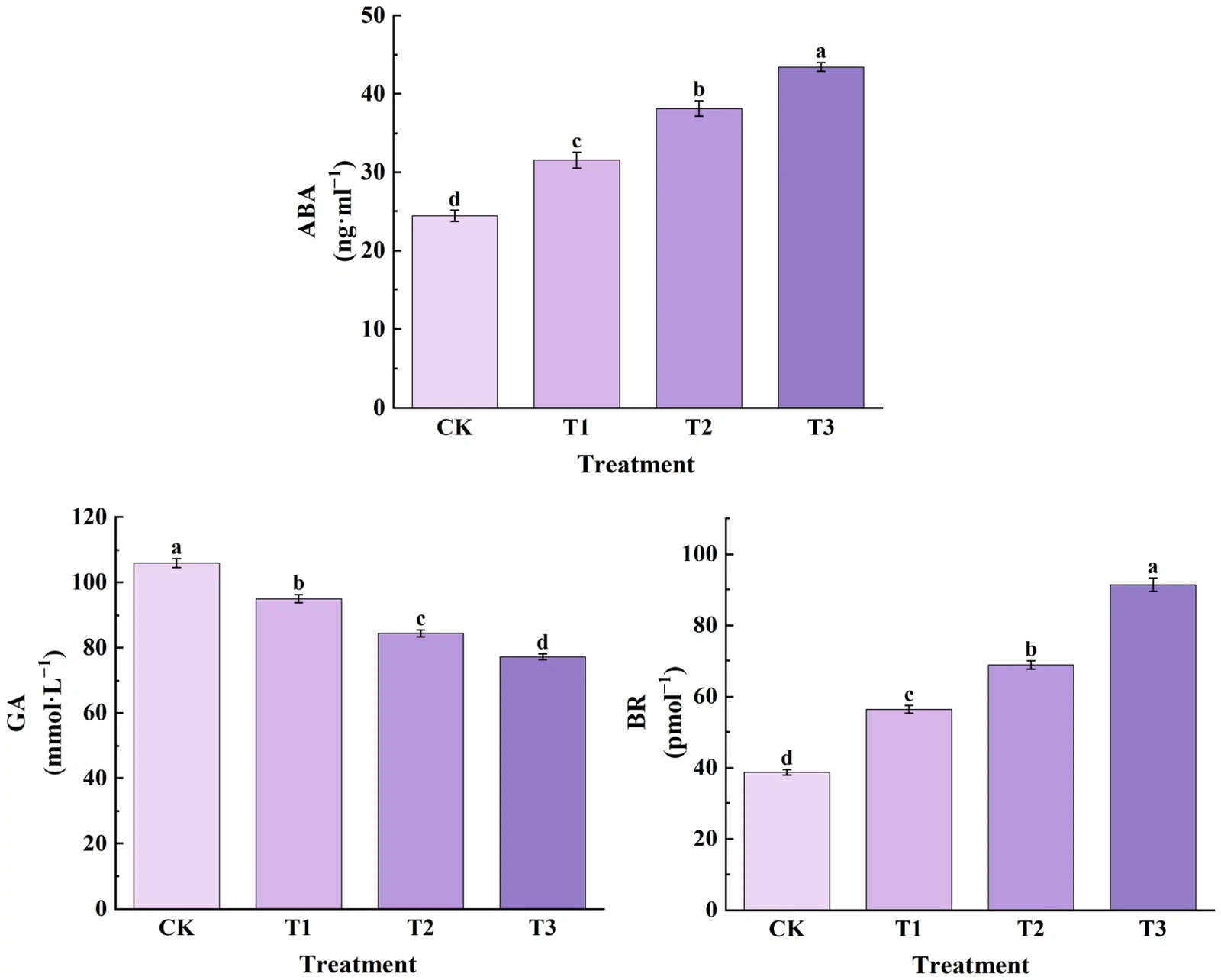
Effects of Alkaline Salt Stress on Hormone Contents in Potato Leaves. Note: CK, 0 mmol/L NaHCO_3_; T1, 20 mmol/L NaHCO_3_; T2, 40 mmol/L NaHCO_3_; and T3, 60 mmol/L NaHCO_3_. Data are presented as the mean ± standard error (SE) (n = 3). Different lowercase letters above the bars indicate significant differences among treatments (*p* < 0.05).

As shown in Figure 3, different levels of alkaline salt stress significantly affected hormone contents in potato, and the measured parameters showed different trends with increasing stress severity. Specifically, with increasing sodium bicarbonate treatment concentration, the contents of ABA and BR in potato plants showed increasing trends, whereas GA content showed a decreasing trend. For ABA content, compared with CK, the different treatments increased by 29.11%, 56.28%, and 78.07%, respectively, whereas GA content decreased by 11.49%, 25.54%, and 37.29%, respectively. For BR content, the increase compared with CK ranged from 45.53% to 135.99% across the different treatments. Therefore, increasing saline-alkali stress severity increased ABA and BR contents and decreased GA content in potato plants. In other words, the increase in ABA and BR contents and the decrease in GA content may contribute to the response of potato plants to alkaline salt stress.

### 2.4. Effects of Alkaline Salt Stress on Key Genes Involved in Lignin and Hormone Biosynthesis in Potato

**Figure 4:**
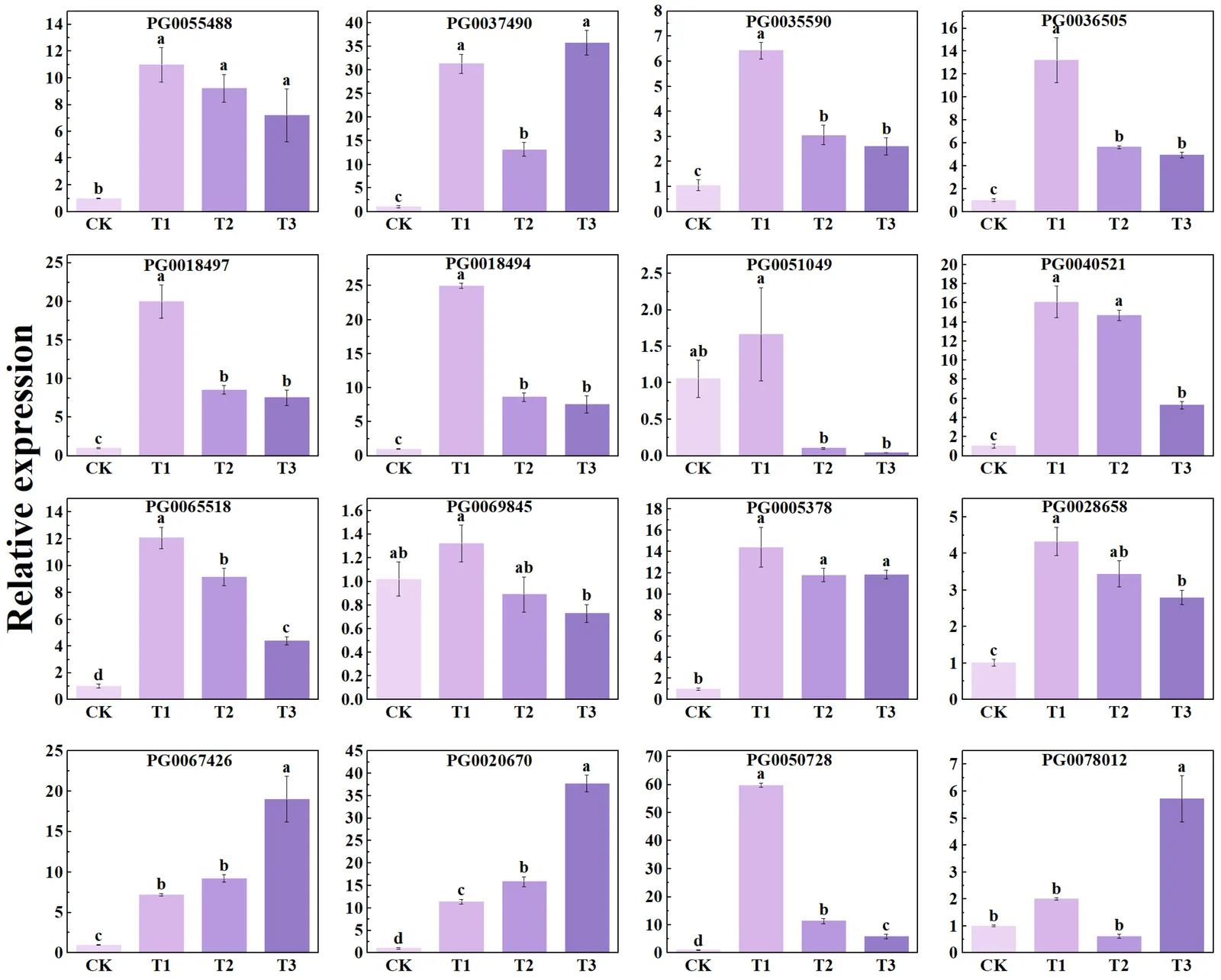
Effects of Alkaline Salt Stress on the Expression of Key Genes Involved in Root Lignin Biosynthesis and Leaf Hormone Signaling in Potato. Note: PG is the abbreviation of PGSC0003DMG40, CK, 0 mmol/L NaHCO_3_; T1, 20 mmol/L NaHCO_3_; T2, 40 mmol/L NaHCO_3_; and T3, 60 mmol/L NaHCO_3_. Data are presented as the mean ± standard error (SE) (n = 3). Different lowercase letters above the bars indicate significant differences among treat-ments (*p* < 0.05).

Lignin plays an important mechanical protective role in plants when they are exposed to abiotic stresses such as saline-alkali stress. Under alkaline salt stress, potato plants increase lignin accumulation by regulating key genes involved in lignin biosynthesis, including *PG0055488*, *PG0037490*, *PG0035590*, *PG0036505*, *PG0018497*, *PG0018494*, *PG0051049*, *PG0040521*, *PG0065518*, thereby enhancing the resistance of potato plants to alkaline salt stress. As shown in Figure 4, the results showed that genes related to the phenylpropanoid pathway and lignin biosynthesis were predominantly upregulated overall, but their response patterns to stress concentration differed among genes. Specifically, PAL, C4H, HCT, C3’H, CAD, and POD genes were generally upregulated compared with CK and showed varying degrees of decline with increasing stress concentration, whereas 4CL showed a trend of first increasing, then decreasing, and then increasing again. In contrast, CCR showed an overall downregulation trend. In addition, alkaline salt stress also significantly affected the expression of key genes involved in the ABA, GA, and BR biosynthetic pathways in potato, including *PG0069845*, *PG0005378*, *PG0028658*, *PG0067426*, *PG0020670*, *PG0050728*, *PG0078012*. Overall, these genes showed different degrees of upregulation or downregulation compared with CK and exhibited distinct concentration-dependent responses.

In summary, alkaline salt stress significantly affected the expression of genes related to lignin biosynthesis and ABA, GA, and BR biosynthesis and signal transduction in potato. Lignin biosynthesis-related genes were predominantly significantly upregulated overall, but different genes showed different response patterns to stress concentration. The expression levels of ABA-, GA-, and BR-related genes were generally higher than those in CK and exhibited different concentration-dependent trends.

### 2.5. Integrated Response Model of Antioxidant Defense, Hormone Signaling, and Lignin Biosynthesis in Potato Under Alkaline Salt Stress

**Figure 5:**
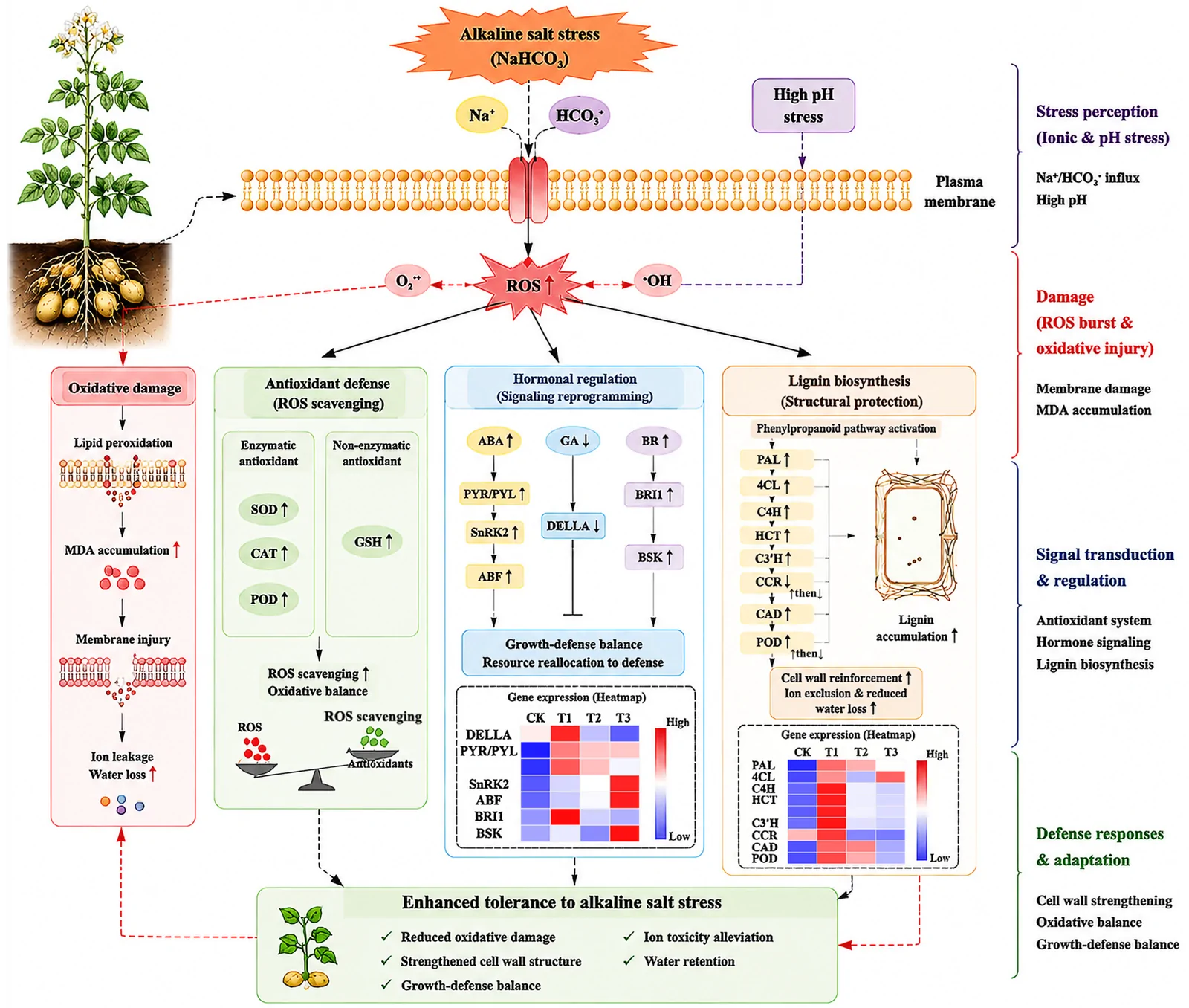
Integrated response model of antioxidant defense, hormone signaling, and lignin biosynthesis in potato under alkaline-salt stress. Note: NaHCO_3_ stress induces the accumulation of Na^+^ and HCO_3_^−^, disrupts pH homeostasis, and promotes reactive oxygen species (ROS) accumulation and oxidative damage. Potato plants respond by regulating the antioxidant system (SOD, CAT, POD, and GSH), hormone signaling pathways (ABA, GA, and BR), and the expression of genes associated with lignin biosynthesis. These responses contribute to ROS scavenging, cell wall reinforcement, and the maintenance of growth–defense balance, ultimately enhancing tolerance to alkaline salt stress. The arrows ↑ and ↓ indicate increases and decreases, respectively. The heatmap shows the relative expression levels of the corresponding genes under different treatments. The proposed model should be presented as a working hypothesis rather than a demonstrated mechanism.

Compared with neutral salt stress, alkaline salt stress is characterized by a relatively high pH stress. As shown in Figure 5, based on the changes in physiological indicators, the antioxidant system, hormone-related genes, and lignin biosynthesis-related genes, an integrated regulatory model of the potato response to alkaline salt stress was constructed. The model shows that with increasing NaHCO_3_ concentration, Na^+^ and HCO_3_^−^ enter plant cells, accompanied by responses such as ROS accumulation, membrane lipid peroxidation, and membrane damage.

In terms of antioxidant defense, SOD activity continuously increased under alkaline salt stress, whereas POD and CAT activities first increased and then decreased. GSH content continuously increased, accompanied by changes in MDA content, indicating that the antioxidant system of potato plants responded markedly to alkaline salt stress. These changes were consistent with those observed in indicators related to ROS scavenging and oxidative damage. In terms of hormone regulation, the model showed that alkaline salt stress involved ABA, GA, and BR related signaling pathways. The expression of ABA-related PYR/PYL (*PG0005378*, *PG0028658*), SnRK2 (*PG0067426*), and ABF (*PG0020670*) genes was generally upregulated. The GA-related DELLA gene (*PG0069845*) first increased and then decreased with increasing stress concentration, showing an overall downregulation trend. The expression of the BR-related BRI1 (*PG0050728*) and BSK (*PG0078012*) genes was generally upregulated. Meanwhile, the hormone signaling-related genes shown in the figure exhibited different response patterns to alkaline salt stress. In terms of lignin biosynthesis, the expression patterns of genes related to the phenylpropanoid pathway changed significantly with increasing stress severity. Among them, the PAL-related gene (*PG0055488*) was significantly upregulated after stress treatment, indicating that the phenylpropanoid pathway was induced and enhanced by alkaline salt stress. The 4CL-related gene (*PG0037490*), C4H-related gene (*PG0035590*), HCT-related gene (*PG0036505*), C3’H-related genes (*PG0018497*, *PG0018494*), CAD-related gene (*PG0040521*), and POD-related gene (*PG0065518*) showed overall upregulation trends, whereas CCR (*PG0051049*) showed an overall downregulation trend. Lignin accumulation changed with the stress treatment.

In summary, potato plants showed obvious responses to alkaline salt stress in terms of oxidative stress, hormone signaling, and lignin biosynthesis. The antioxidant system, ABA/GA/BR-related signaling pathways, and genes related to the phenylpropanoid pathway and lignin biosynthesis all exhibited changes to varying degrees.

## 3. Discussion

Soil salinization and alkalization is a typical abiotic stress that affects crop growth and yield. Alkaline salts represented by NaHCO_3_ impose both ionic toxicity and high-pH stress, and their effects on plants are generally more severe than those of neutral salt stress represented by NaCl [39,40]. Both conventional salt stress and alkaline salt stress can induce ion imbalance, reactive oxygen species accumulation, and changes in the antioxidant system, representing common plant responses to salt stress [41]. However, NaHCO_3_ stress also involves high pH and the effects of HCO_3_^−^, which may lead to physiological responses distinct from those induced by conventional salt stress [42]. As NaCl and pH-only controls were not included in the present study, it remains difficult to distinguish general responses to salt stress from responses specific to alkaline stress. Further investigations are therefore needed to clarify these differences. Alkaline salt stress not only causes disruption of cellular ion balance, osmotic stress, and oxidative damage, but also affects secondary metabolic processes by interfering with endogenous hormone contents, thereby affecting plant growth and development at multiple levels [43,44]. In particular, as shown in Figure S1, saline-alkali stress has a significant adverse effect on plant growth in potato production [45]. In this study, the responses of potato to NaHCO_3_-induced alkaline salt stress were further investigated by analyzing changes in the antioxidant system, root lignin accumulation, and endogenous hormones such as ABA, GA, and BR, together with the expression of genes related to lignin biosynthesis and hormone signaling. The results showed that with increasing NaHCO_3_ concentration, the degree of oxidative stress in potato gradually increased, with SOD activity and MDA and GSH contents continuously increasing, whereas POD and CAT activities first increased and then decreased. Meanwhile, lignin continuously accumulated in the roots, ABA and BR contents increased, and GA content decreased. The expression of genes related to the phenylpropanoid pathway and ABA, GA, and BR also changed accordingly, indicating that potato responds to alkaline salt stress through the combined regulation of antioxidant defense, cell wall structural adjustment, and hormone signaling.

Oxidative stress is an important physiological process contributing to cell damage caused by saline-alkali stress [46]. After high concentrations of Na^+^ and HCO_3_^−^ enter root cells, they may disrupt the intracellular redox balance and promote ROS accumulation. Excessive ROS can attack cell membranes and induce membrane lipid peroxidation, while MDA, an important product of membrane lipid peroxidation, can reflect the degree of cell membrane damage to some extent [47]. In this study, MDA content continuously increased with increasing NaHCO_3_ concentration, with the maximum increase reaching 87.07%, indicating that the degree of membrane lipid peroxidation in potato continuously increased with increasing stress intensity. This result is similar to previous findings showing increased MDA accumulation in salt-tolerant wheat under salt stress conditions [48]. Therefore, oxidative damage induced by alkaline salt stress may be an important early physiological change in potato in response to stress. Facing continuously increasing oxidative pressure, plants commonly maintain intracellular redox balance through the combined action of enzymatic and non-enzymatic antioxidant systems [49]. In this study, SOD activity continuously increased with increasing NaHCO_3_ concentration, indicating that potato plants may promote the conversion of superoxide anions to H_2_O_2_ by increasing SOD activity. Meanwhile, POD and CAT activities both first increased and then decreased, reaching relatively high levels under 40 mmol/L NaHCO_3_ treatment. This pattern indicated that under low to moderate stress levels, potato plants were able to timely remove excess ROS by enhancing antioxidant enzyme activities, whereas under the high-concentration treatment of 60 mmol/L NaHCO_3_, the decrease in POD and CAT activities suggested that as stress further intensified, oxidative pressure may have gradually exceeded the regulatory capacity of the enzymatic defense system [50]. As shown in Figure 1, SOD activity continued to increase under the highest NaHCO_3_ concentration, whereas POD and CAT activities decreased. This pattern may be associated with the differential responses of antioxidant enzymes to stress. SOD primarily catalyzes the conversion of superoxide radicals (O_2_^−^·) into H_2_O_2_, while CAT and certain POD enzymes are involved in H_2_O_2_ scavenging. Therefore, the continued increase in SOD activity, accompanied by decreases in POD and CAT activities, may indicate that the plants maintained an enhanced SOD response to stress, whereas the activities of H_2_O_2_-scavenging enzymes did not increase accordingly. The declines in POD and CAT activities under more severe stress may be associated with enzyme inhibition, oxidative damage, or limitations in the plant’s antioxidant regulatory capacity [51]. In contrast to enzymatic defense, GSH content continuously increased with increasing stress concentration and remained on an increasing trend even under high-concentration stress. GSH can participate in free radical scavenging and maintain the intracellular reducing environment. Therefore, its continuous increase may help buffer the oxidative pressure caused by the decline in enzymatic antioxidant capacity under high-intensity stress, which is consistent with previous studies showing the involvement of GSH in antioxidant defense under various plant stress conditions [52,53]. Taken together, the antioxidant system of potato showed a clear stress intensity-dependent response. Under low to moderate stress levels, antioxidant defense was synchronously activated with increasing stress intensity, whereas under high-intensity stress, the activities of some antioxidant enzymes began to decline, while GSH remained at a relatively high level, indicating that different antioxidant components may have a certain complementary effect. It can therefore be inferred that potato plants are able to alleviate ROS-induced oxidative damage through the coordinated action of enzymatic and non-enzymatic defenses within a certain range of stress. However, as stress severity further increases, the overall defense capacity of potato plants may gradually approach their tolerance threshold.

In addition to antioxidant defense, endogenous hormones are also important regulatory systems involved in plant perception and responses to adverse environmental conditions and can coordinate resource allocation between growth and defense through intracellular signal transduction [54]. This study focused on abscisic acid (ABA), gibberellin (GA), and brassinosteroids (BR) based on their roles in plant stress responses and the existing research evidence. In our previous study, transcriptomic analysis revealed substantial changes in genes associated with these three hormone signaling pathways under alkaline salt stress, which provided the basis for selecting ABA, GA, and BR for further investigation in the present study. ABA is an important hormone involved in plant responses to salt stress, contributing to water balance and the regulation of antioxidant defense. GA is closely associated with plant growth and development as well as stress adaptation [41]. Therefore, these two hormones can help elucidate growth regulation and defense responses in potato under stress conditions. In contrast, the role of BR in potato responses to alkaline salt stress has received relatively little attention [30]. Previous studies have shown that exogenous application of 24-epibrassinolide (EBR) can enhance potato tolerance to alkaline salt stress by improving antioxidant defense and photosynthetic performance. Accordingly, this study further investigated BR content and the expression of related genes to provide additional insights into potato responses to alkaline salt stress [55]. In this study, the contents of ABA, GA, and BR and the key genes involved in their related signaling pathways changed markedly, indicating that all three hormones jointly participated in the response of potato to alkaline salt stress. ABA is an important signaling molecule involved in plant responses to abiotic stress. In this study, ABA content continuously increased with increasing NaHCO_3_ concentration, while the expression of genes related to PYR/PYL, SnRK2, and ABF was generally upregulated, indicating that the ABA signaling pathway may have been induced by stress. Consistent with the study by Jiajie Wang et al. on salt stress in maize, the regulatory mechanism involves the binding of ABA to PYR/PYL receptors, which releases the inhibition of SnRK2 by PP2C. Activated SnRK2 subsequently regulates downstream ABF transcription factors through phosphorylation, thereby initiating the expression of various stress-responsive genes [56]. Therefore, the simultaneous changes in ABA content and related signaling genes in the present study indicated that ABA may serve as an important signaling node in the response of potato to alkaline salt stress and participate in the regulation of downstream defense processes. In contrast to ABA, GA content continuously decreased with increasing stress severity, while the related DELLA gene showed an overall downregulation trend. GA is an important hormone that promotes plant growth, and a decrease in GA levels generally favors the accumulation of DELLA proteins in cells. DELLA proteins can inhibit certain growth processes, thereby enabling plants to reduce growth investment under adverse conditions and allocate more limited resources to defense processes. Consistent with the study by Jia Dong et al. in soybean, their results showed that DELLA is involved in hormone regulation and stress responses. Therefore, the decrease in GA levels observed in the present study may reflect the regulation of the balance between growth and defense in potato under alkaline salt stress [57]. Meanwhile, changes in the expression of DELLA-related genes may also reflect feedback regulation of the degree of growth inhibition in potato plants, thereby preventing growth from being completely inhibited under adverse conditions. BR showed a trend similar to that of ABA. In the present study, BR content increased markedly with increasing stress concentration, and the expression of BRI1- and BSK-related genes was generally upregulated, indicating that BR signaling may participate in the response to alkaline salt stress. Consistent with previous research on alkaline salt stress in potato, BR can alleviate saline-alkali stress-induced damage by enhancing cell membrane stability and coordinately increasing antioxidant enzyme activities [58]. BR can also interact with ABA and GA signaling through cross-regulation to jointly coordinate plant growth and defense. Therefore, ABA, GA, and BR may jointly participate in the adaptation of potato to alkaline salt stress through different but interconnected regulatory mechanisms. In addition to hormone regulation, remodeling of the root cell wall structure is also an important defense strategy for plants in response to saline-alkali stress [59]. As an important structural component of the cell wall, lignin accumulation can enhance cell wall mechanical strength, restrict the transport of harmful ions, and reduce water loss, thereby improving the ability of roots to adapt to adverse environmental conditions [60]. Jiancai Xiao et al. reported in a study of salt tolerance in licorice that plants can improve root stress resistance by promoting lignin biosynthesis to form a physical barrier, which is consistent with the results of the present study [61]. In this study, lignin content in potato roots continuously increased with increasing NaHCO_3_ concentration, with the maximum increase reaching 98.19%, indicating that alkaline salt stress can promote lignin accumulation in potato roots, which may participate in stress defense by strengthening the cell wall structure.

Further analysis of the expression of genes related to the phenylpropanoid pathway showed that lignin accumulation was closely associated with the coordinated responses of multiple metabolic nodes. In this study, genes related to PAL, C4H, and 4CL were all significantly upregulated, indicating that alkaline salt stress can promote the upstream reactions of the phenylpropanoid pathway and provide precursor substances for lignin biosynthesis. Among them, the expression level of the PAL-related gene slightly decreased with increasing stress concentration, suggesting that this pathway may be inhibited to some extent under high-intensity stress. Peijie Wang et al. reported that PAL, C4H, and 4CL are important key enzymes in the phenylpropanoid pathway and lignin biosynthesis and play important roles in lignin formation [62]. In the present study, genes related to HCT, C3’H, and CAD were also induced by alkaline salt stress, indicating that the middle and downstream steps of the phenylpropanoid pathway maintained a strong response capacity, which was conducive to the further formation of lignin monomers. In contrast, CCR-related genes showed an overall downregulation trend and increased slightly only under low-concentration stress, suggesting that CCR may be an important sensitive node in the phenylpropanoid pathway under high-concentration stress, and its suppressed expression may limit the further synthesis of lignin monomers. Aiting Zhou et al. also reported that HCT, C3’H, and CAD participate in the hydroxylation modification and reduction reactions involved in lignin monomer formation and are important functional nodes in lignin biosynthesis [63]. In addition, POD participates in both H_2_O_2_ scavenging and the oxidative polymerization of lignin monomers. In this study, the expression level of the POD-related gene gradually decreased with increasing stress concentration, which was generally consistent with the trend of POD enzyme activity, which first increased and then decreased. This suggests that the decrease in POD-related transcription and enzyme activity under high stress conditions may limit the further polymerization of lignin monomers. Tingting Xu et al. reported in poplar that POD at the terminal stage of lignin biosynthesis participates in the oxidative polymerization of lignin monomers and plays an important role in final lignin deposition [64]. Therefore, although the expression of some phenylpropanoid pathway genes decreased under high-concentration stress, root lignin content continued to increase, indicating that lignin accumulation may result from the integrated regulation of the entire phenylpropanoid metabolic network rather than being determined by a single gene.

Based on the present study and relevant literature, the lignin biosynthesis-related genes PAL, C4H, 4CL, HCT, C3′H, CCR, and CAD are hypothesized to be primarily involved in phenylpropanoid metabolism and lignin monomer formation, whereas POD may participate in the oxidative polymerization and deposition of lignin monomers. (Figure S2). Among the hormone-related genes, PYR/PYL, SnRK2, and ABF are involved in ABA signal perception, transduction, and transcriptional regulation, respectively; DELLA participates in GA-mediated growth regulation, while BRI1 and BSK are involved in BR signal perception and transduction, respectively. These genes may collectively contribute to potato adaptation to NaHCO_3_ stress by regulating lignin biosynthesis and hormone signaling responses.

By integrating the results of the antioxidant system, hormone regulation, and lignin metabolism, it can be seen that the response of potato to NaHCO_3_-induced alkaline salt stress shows an obvious multilevel coordination. Alkaline salt stress may first cause changes in ion homeostasis and redox balance in the roots, promoting ROS accumulation and membrane lipid peroxidation, as indicated by the continuous increase in MDA content. Subsequently, endogenous hormones such as ABA, GA, and BR undergo dynamic changes and participate in stress signal transduction and the regulation of the growth–defense balance through related signaling pathways. Meanwhile, these changes are accompanied by changes in the expression of genes related to the phenylpropanoid pathway, thereby promoting lignin accumulation in the roots. In addition, antioxidant defense and structural defense are not independent of each other. POD participates in both ROS scavenging and the oxidative polymerization of lignin monomers and may therefore serve as an important connection point between the two defense processes. It can therefore be seen that the response of potato to alkaline salt stress involves multiple processes, including antioxidant defense, hormone regulation, and lignin biosynthesis. Under low to moderate stress levels, potato plants can alleviate stress-induced damage by enhancing antioxidant capacity, regulating hormone-related processes, and promoting lignin accumulation. As stress intensity increases, the responses of some antioxidant enzymes and genes related to the phenylpropanoid pathway gradually weaken, indicating that the defense capacity of potato plants is affected to some extent under high-intensity alkaline salt stress. Overall, antioxidant defense, endogenous hormone regulation, and root lignin accumulation together constitute a multilevel response system of potato to alkaline salt stress, providing a basis for further understanding the physiological mechanisms underlying salt and alkali tolerance in potato.

## 4. Materials and Methods

### 4.1. Experimental Design

The potato cultivar ‘Atlantic’ used in this experiment was pre-basic seed potato, provided by Dingxi Academy of Agricultural Sciences, Gansu Province, China. The potassium sulfate fertilizer (K_2_O 52%, 20 kg per bag) used in this study was imported from Russia. Other fertilizers including urea, superphosphate, and sodium bicarbonate (NaHCO_3_) were purchased from Lanzhou Xinyuan Biotechnology Co., Ltd., with a specification of 500 g per box.

The pot experiment was carried out at Gansu Agricultural University (No. 1 Yingmen Village, Anning District, Lanzhou, China; 36.0908° N, 103.6989° E) to investigate the effects of alkaline salt stress on potato. Planting pots with a diameter of 30 cm and a height of 35 cm were used, and drainage holes were drilled at the bottom of each pot. The growth substrate was a mixture of vermiculite and perlite at a volume ratio of 4:1, and carbendazim was applied to fully disinfect the substrate. The treated substrate was filled into pots to a height of 26 cm. Twenty-four hours before sowing, the substrate was fully watered, and basal fertilizer was uniformly applied simultaneously. Seed tubers with intact, healthy sprouts and free of diseases and insect pests were carefully selected. After air drying, one seed tuber was sown in each pot with the sprout facing upward. Alkaline salt stress treatment was initiated after potato seedling emergence.

Four NaHCO_3_ concentration treatments were set: CK: 0 mmol/L (pH: 6.781, EC: 806 μS/cm), T1: 20 mmol/L (pH: 7.530, EC: 862 μS/cm), T2: 40 mmol/L (pH: 7.835, EC: 879 μS/cm) and T3: 60 mmol/L (pH: 8.125, EC: 1012 μS/cm). Twelve pots of plants were used for each treatment, with three independent biological replicates. For the first stress treatment, each pot was fully irrigated with NaHCO_3_ solution of the corresponding concentration. Afterwards, NaHCO_3_ solution of the same concentration was applied every 7 days. Weeds were regularly removed throughout the experiment to guarantee normal plant growth and reduce non-experimental interference. Samples were collected on day 28 after stress treatment, corresponding to the full-flowering stage of potato. Leaf and root samples were collected separately from plants in each biological replicate. The leaves were used for the analysis of antioxidant enzyme activities, oxidative damage-related parameters, endogenous hormone levels, and gene expression, whereas the roots were used to determine lignin content. All samples were labeled individually to avoid pooling material from different biological replicates. Immediately after collection, the samples were flash-frozen in liquid nitrogen and subsequently stored at −80 °C. 

### 4.2. Determination of Indicators and Methods

#### 4.2.1. Antioxidant and Oxidative Damage-Related Indicators in Potato

Antioxidant and oxidative damage indicators were determined using biochemical assay kits from Beijing Solarbio Science & Technology Co., Ltd. (Beijing, China). SOD and POD activities were measured with the SOD Activity Assay Kit (BC0175) and POD Activity Assay Kit (BC0095), respectively, following spectrophotometric protocols. CAT activity was assayed using the CAT Activity Assay Kit (BC4785) via the ammonium molybdate method at 405 nm. MDA content was determined with the MDA Content Assay Kit (BC0025) based on the thiobarbituric acid (TBA) reaction. GSH content was quantified using the GSH Content Assay Kit (BC1175) by the DTNB method at 412 nm.

#### 4.2.2. Determination of Lignin Content and Endogenous Hormone Contents in Potato Roots

Lignin and endogenous hormones (except BR) in potato roots were measured using Solarbio assay kits (Beijing, China). Lignin content was determined with the Lignin Content Assay Kit (BC4200) using ultraviolet spectrophotometry. Root samples were oven-dried to constant weight, and 5 mg dry sample was weighed for extraction and detection according to the kit instructions; absorbance was recorded at 280 nm to calculate lignin content. ABA and GA contents were detected using hormone assay kits BC728H and BC729H, respectively. Measurements were performed following the manufacturer’s instructions, and hormone concentrations were calculated against standard curves. BR content was determined using the kit HB024-Pt from Shanghai Hengyuan Biotechnology Co., Ltd. (Shanghai, China).

#### 4.2.3. RNA Extraction, Reverse Transcription, and Real-Time Quantitative Polymerase Chain Reaction

Potato leaf samples were collected for total RNA extraction using the SteadyPure Plant RNA Extraction Kit (AG21019). RNA integrity and concentration were checked with a Pultton P 100+ ultra-micro spectrophotometer (OSIC HOLDINGS, Beijing, China). The HiScript III 1st Strand cDNA Synthesis Kit (+gDNA wiper) (R312) was used for reverse transcription to generate first-strand cDNA. RT-qPCR primers were synthesized by Sangon Biotech (Shanghai, China) Co., Ltd. (Table 1). qPCR was performed on a real-time fluorescence quantitative PCR instrument using the SYBR Green Pro Taq HS Premix qPCR Kit II (AG11702). Each 20 μL qPCR reaction contained 10 μL of 2× SYBR Green Pro Taq HS Premix II, 1 μL of forward primer, 1 μL of reverse primer, 1 μL of cDNA template, and 7 μL of RNase-free water. Relative gene expression was calculated by the 2^−ΔΔCt^ method [65]. *StEF-1α* was used as the reference gene, and three independent technical replicates were set for each reaction. Three independent biological replicates were included for each treatment, with three technical replicates for each biological replicate. Samples from different biological replicates were collected and processed independently, including RNA extraction, cDNA synthesis, and qPCR analysis.

The lignin biosynthesis-related genes analyzed in this study were primarily selected from previous transcriptomic data generated by our research group. Based on their functional annotations in the phenylpropanoid metabolism and lignin biosynthesis pathways, genes associated with PAL, C4H, 4CL, HCT, C3′H, CCR, CAD, and POD were selected for qPCR analysis (Gene ID, corresponding gene name, and abbreviation are shown in Table S1). Genes involved in plant hormone signal transduction were also selected based on the transcriptomic data generated by our research group and their functional annotations in the ABA, GA, and BR signaling pathways. Representative candidate genes were selected for qPCR analysis to investigate their expression responses to NaHCO_3_ stress.

#### 4.2.4. Data Analysis

Microsoft Excel 2021 (Microsoft Corporation, Redmond, WA, USA) was used for data sorting. Origin 9.0 (OriginLab Corporation, Northampton, MA, USA) was adopted to plot figures. SPSS 27.0 (IBM Corporation, Armonk, NY, USA) was used for one-way analysis of variance (ANOVA), multiple comparisons and correlation analysis. One-way analysis of variance (ANOVA) was used to assess differences among treatments. When significant differences were detected, multiple comparisons were performed using the least significant difference (LSD) test or Duncan’s multiple range test, as appropriate. Differences were considered statistically significant at *p* < 0.05.

## 5. Conclusions

In this study, potato cultivar ‘Atlantic’ was used as the experimental material, and different concentrations of sodium bicarbonate were applied to establish alkaline salt stress treatments. The results showed that with increasing stress severity, oxidative damage to potato cell membranes continuously increased, while the antioxidant system exhibited a pattern of initial activation followed by physiological imbalance. Meanwhile, potato plants enhanced root lignin accumulation by upregulating key genes involved in the phenylpropanoid pathway, thereby strengthening cell wall structure and alleviating stress-induced damage. Endogenous hormone levels also changed significantly under alkaline salt stress; increased abscisic acid and brassinosteroid contents and decreased gibberellin content coordinated the resource allocation between plant growth and stress defense. Antioxidant defense, lignin-based structural defense, and hormone signaling regulation interacted and synergistically functioned to form a multilevel adaptive mechanism of potato response to alkaline salt stress. These findings provide a theoretical reference for optimizing potato cultivation in saline-alkali soils and breeding salt- and alkali-tolerant potato varieties.

## Figures and Tables

**Table 1:** Gene IDs and primer sequences for the genes used for qPCR verification.

Gene ID	Forward Primer	Reverse Primer
PGSC0003DMT400055488	TTGTCCCTCTGTCCTACA	CAACTCGAAAAATCCACC
PGSC0003DMT400037490	GCTCATTATCACACAAGA	GTAAGTAGCCCTCAGACG
PGSC0003DMT400035590	ACTCCACACCCCAATACC	CCTCCTCGAGAAACCTCT
PGSC0003DMT400036505	TTCTACACCAAAGATGCT	TTTCTGATATGAGGCAAT
PGSC0003DMT400018497	GCACCCTCCAACTCCCCT	CACGACCTAACGCCCACA
PGSC0003DMT400018494	GCACCCTCCAACTCCCCT	ATCACGACCTAACGCCCA
PGSC0003DMT400051049	GGCTGTGACGGGGTTTTC	CGGTTGGGGTCCATGTAT
PGSC0003DMT400040521	TCCATTGCTTATGTTGAT	TATGTTATGCTTTGCTGC
PGSC0003DMT400065518	AATCAGACGGAAAAACTA	ATCAGCACAAGAAACAAC
PGSC0003DMT400069845	AGATAGGTAGTGGTATGC	ACAATGTTGAAAGAAAAG
PGSC0003DMT400005378	TCATCAGGAAGCAGAAGA	TCGGTAGGAGTGGAACTC
PGSC0003DMT400028658	TACCCGATTCTGTTTCCC	AATGCTTATACGCCTGCG
PGSC0003DMT400067426	TGGAGAACTTTTTGGTAG	TATTTTTAAACGTGGTGA
PGSC0003DMT400020670	CAGTTCCTTATGTGTTTA	GCTGACTCTCTATTTTTT
PGSC0003DMT400050728	GTATCCTCTCCCCCTACC	CCTCCTCTTCTTCGTCTC
PGSC0003DMT400078012	TGAAACCACCTCACCCAC	GCCCACATCTGAAACGAA

## Data Availability

The datasets generated and analyzed during the current study are available in the NCBI repository, https://www.ncbi.nlm.nih.gov/sra/?term=SRP298983 (accessed on 16 January 2026), the accession number is SRP298983. The datasets used and analyzed in the current study are available from the corresponding author on reasonable request.

## Supplementary Materials

The following supporting information can be downloaded at https://www.mdpi.com/article/10.3390/plants15213377/s1, Figure S1: Effects of alkaline salt stress on potato plants; Figure S2: Lignin biosynthesis pathway; Table S1: Gene ID, corresponding gene name, and abbreviation.

## Abbreviations

The following abbreviations are used in this manuscript:

SOD	superoxide dismutase
POD	peroxidase
CAT	catalase
MDA	malondialdehyde
GSH	glutathione/reduced glutathione
ABA	abscisic acid
GA	gibberellin
BR	brassinosteroid
ROS	reactive oxygen species
PAL	phenylalanine ammonia-lyase
4CL	4-coumarate-CoA ligase
C4H	cinnamate 4-hydroxylase
HCT	hydroxycinnamoyl-CoA:shikimate/quinate hydroxycinnamoyltransferase
C3’H	p-coumaroyl shikimate 3’-hydroxylase
CCR	cinnamoyl-CoA reductase
CAD	cinnamyl alcohol dehydrogenase
DELLA	DELLA protein
PYR/PYL	pyrabactin resistance 1/PYR1-like
SnRK2	sucrose non-fermenting 1-related protein kinase 2
ABF	ABA-responsive element binding factor
BRI1	brassinosteroid insensitive 1
BSK	brassinosteroid signaling kinase
