# Supplementary material for: Antioxidant Defense, Lignin Biosynthesis and Hormone Signaling Coordinately Mediate the Response of Solanum tuberosum L. to Alkaline Salt Stress
Source: Plants (Basel). 2026 Nov 1;15(21):3377. doi: 10.3390/plants15213377 (PMC13765656; doi:10.3390/plants15213377)
Supplement: Supplementary file 1 [file plants-15-03377-s001.zip › Table S1.pdf]

Table S1. Gene ID, corresponding gene name, and abbreviation.

Abbreviation	Enzyme / protein (full name)	Gene ID
PAL	Phenylalanine ammonia-lyase	PG0055488
4CL	4-coumarate:CoA ligase	PG0037490
C4H	Cinnamate 4-hydroxylase	PG0035590
HCT	Hydroxycinnamoyl-CoA:shikimate/quinate hydroxycinnamoyltransferase	PG0036505
C3′H	p-coumaroyl shikimate 3'-hydroxylase	PG0018494
C3′H	p-coumaroyl shikimate 3'-hydroxylase	PG0018497
CCR	Cinnamoyl-CoA reductase	PG0051049
CAD	Cinnamyl alcohol dehydrogenase	PG0040521
POD	Peroxidase (guaiacol peroxidase)	PG0065518
DELLA	DELLA protein	PG0069845
PYR/PYL (RCAR)	Pyrabactin resistance 1 / PYR1-like (regulatory component of ABA receptor, RCAR)	PG0005378
PYR/PYL (RCAR)	Pyrabactin resistance 1 / PYR1-like (regulatory component of ABA receptor, RCAR)	PG0028658
SnRK2	Sucrose non-fermenting 1-related protein kinase 2	PG0067426
ABF	ABA-responsive element binding factor	PG0020670
BRI1	Brassinosteroid-insensitive 1	PG0050728
BSK	Brassinosteroid signaling kinase	PG0078012
